# Idiopathic intracranial hypertension in patients with cerebral small vessel disease: A case report

**DOI:** 10.1097/MD.0000000000032639

**Published:** 2023-01-06

**Authors:** Wei Liu, Longbin Jia, Lina Xu, Fengbing Yang, Hongjiang Cheng, Huimin Li, Jing Hou, Dandan Zhang, Yan Liu

**Affiliations:** a Department of Neurology, Jincheng People’s Hospital, Jincheng, China; b Changzhi Medical College, Changzhi, China.

**Keywords:** case report, cerebral small vessel disease (CSVD), glymphatic system, idiopathic intracranial hypertension (IIH), transverse sinus stenosis (TSS)

## Abstract

Idiopathic intracranial hypertension (IIH) is a clinical syndrome characterized by increased intracranial pressure (ICP) without any identifiable cause. However, restrictions of cerebrospinal fluid absorption from the cerebral venous system, the glymphatic system overflow, and the cerebrospinal fluid’s lymphatic pathways may be involved in the pathophysiology of IIH. Furthermore, an impaired glymphatic system is also implicated in the initiation and progression of cerebral small vessel disease (CSVD). Here, we reported a case of CSVD with concomitant IIH, possibly associated with the brain’s glymphatic and lymphatic system dysfunction.

**Case concern:**

A 39-year-old male presented with worsening headaches over the bilateral parietal areas during the past year and nausea for 2 days. Fundus examination revealed bilateral papilledema and lumbar puncture suggestive of elevated ICP, laboratory results showed hyperhomocysteinemia and mutation of methylenetetrahydrofolate reductase C677T. On magnetic resonance imaging, subcortical small infarct, white matter lesions, lacunes, enlarged perivascular spaces and dilatation of the optic nerve sheaths was detected, and right transverse sinus stenosis and a hypoplastic left sinus were showed on contrast-enhanced magnetic resonance venography

**Diagnosis:**

The diagnoses of IIH, CSVD, transverse sinus stenosis, and hyperhomocysteinemia were performed.

**Intervision and outcomes:**

The patient received antihypertensive, antiplatelet, anti-atherosclerotic, and homocysteine-lowering therapies. Finally, the patient’s symptoms remised, and the increased ICP returned to normal; however, the bilateral TSS persisted after 3 months of follow-up.

**Conclusions:**

In this case, we speculate that the normal glymphatic outflow pathway may serve as a compensatory mechanism for regulating increased ICP in patients with bilateral venous sinus obstruction, indicating impaired venous outflow pathway, possibly associated with dysfunction of the glymphatic and lymphatic systems in patients with CSVD.

## 1. Introduction

Idiopathic intracranial hypertension (IIH) is characterized by symptoms of increased intracranial pressure (ICP) without an apparent cause.^[1,2]^ Common clinical presentations of IIH include headaches, visual loss, pulsatile tinnitus, and back and neck pain. Neuroimaging IIH profiles include an empty or partially empty sella, distended optic nerve sheath, tortuosity of the optic nerves, posterior displacement of the pituitary stalk, flattening of the posterior globe, bilateral transverse sinus stenosis (TSS), and enlargement of Meckel cave.^[3–5]^ Although the pathogenesis of IHH remains unknown, some possible causal and associated factors exist, such as obesity, female sex, hormones, rapid weight gain, anemia, obstructive sleep apnea, and secondary diseases.^[1,2,6]^ In addition, recent discoveries of the glymphatic and lymphatic systems of the brain may provide new insights into IIH.^[7]^

In the brain, the glymphatic system, a fluid transport system that uses the perivascular network formed by astroglia cells, facilitates fluid clearance.^[8]^ In contrast, the lymphatic system facilitates waste clearance and fluid drainage.^[9]^ Recent studies have demonstrated that impairment of the glymphatic system is associated with various neurological diseases, including IIH and cerebral small vessel disease (CSVD). CSVD is a group of diseases that primarily distresses the small blood vessels in the brain, including the small arteries, capillaries, and veins,^[10]^ manifesting as recent subcortical small infarct, white matter lesions, lacunes, cerebral microbleeds, and enlarged perivascular spaces (EPVS) on neuroimaging.^[11]^ Although some pathophysiological mechanisms of CSVD have been confirmed, including blood-brain barrier dysfunction, decreased cerebral blood flow, vessel stiffening, inflammation, and interstitial fluid (ISF) drainage dysfunction,^[12]^ our poor understanding of the potential mechanisms of small vessel disease requires improvement. In addition, recent research has illustrated the role of glymphatic system impairment in CSVD. Here, we described a patient with CSVD with IIH and TSS that returned to normal ICP after controlling for cerebrovascular risk factors associated with the dysfunction of the glymphatic and lymphatic systems in the brain. However, TSS still existed in an attempt to explore the impact of the glymphatic and lymphatic systems in IIH and CSVD.

## 2. Case report

**Figure 1.**
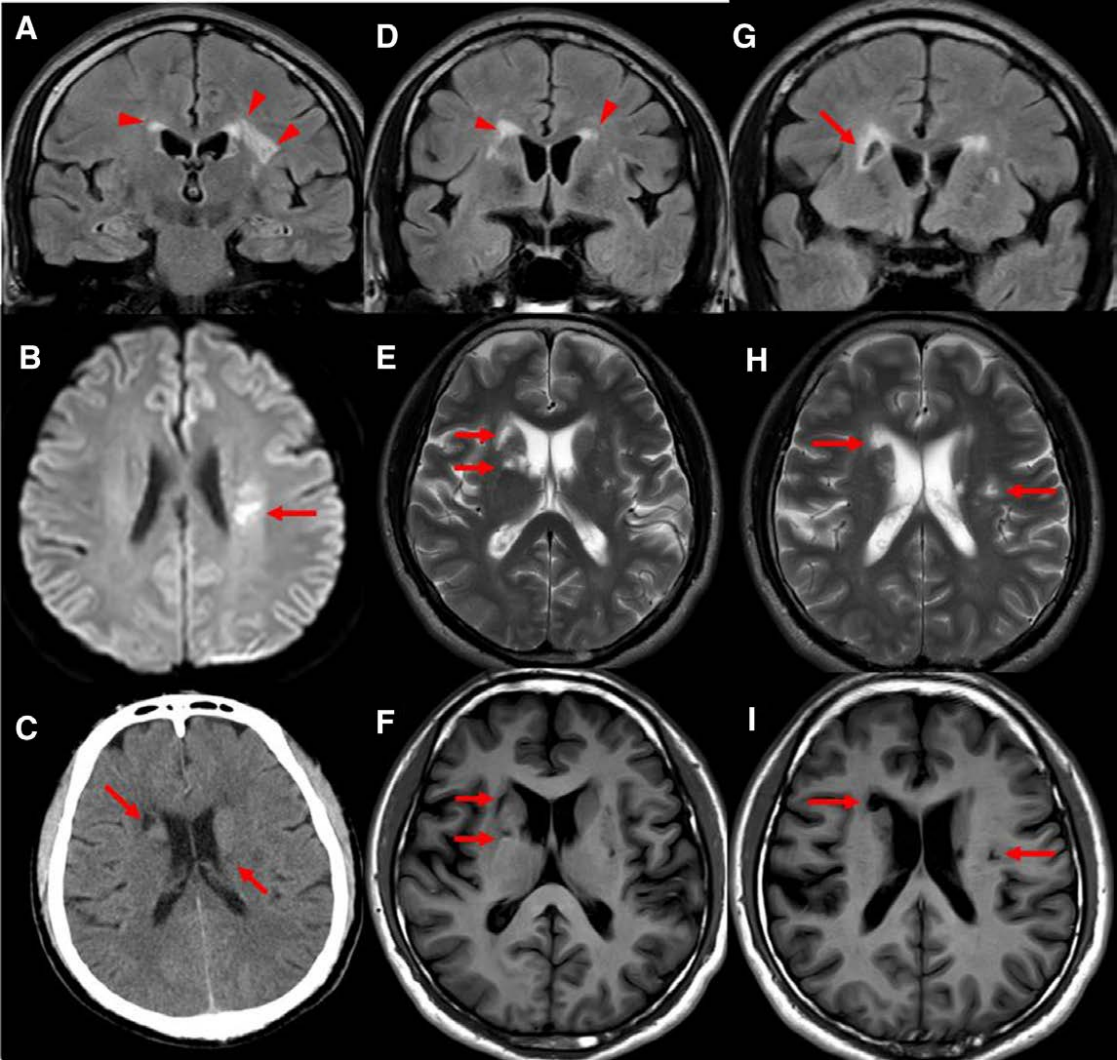
The CT scanning and MRI map during hospitalization. (A and D) white matter lesions in periventricle with hyperintensities on FLAIR (red arrow heads). (C) The cerebral infarctions on CT (red arrows). (B) Recent infarct in the left centrum semiovale on DWI (a red arrow); (E–I) Lacune of presumed vascular origin surrounding ventricle on T2WI, T1WI and FLAIR image (red arrows). CT = computed tomography, DWI = , FLAIR = fluid-attenuated inversion recovery, MRI = magnetic resonance imaging, T1WI = T1-weight, T2WI = T2-weight.

A 39-year-old male presented to the outpatient department of our hospital with a complaint of worsening acute headache for the past 1 year and nausea for 2 days. The headache was localized in the bilateral parietal areas, with mild or moderate intensity; he was not on pain-relieving medicines. His headache had been accompanied by nausea and bilateral blurred vision for the past 2 days; he denied a history of similar headaches. However, the patient had a history of hypertension for 5 years and a family history of hypertension, with no abnormal dietary habits, including smoking or drinking. Physical examination on admission revealed a blood pressure of 185/95 mm Hg and bilateral papilledema (Fig. 6A and B); the rest of the physical examination was normal. The brain’s plain computed tomography (CT) was performed initially, and the results showed cerebral infractions surrounding the ventricles (Fig. 1C). When the clinical profile and results of the CT and fundus examinations were combined, we suspected intracranial hypertension (IH) and cerebrovascular disease.

**Figure 2.**
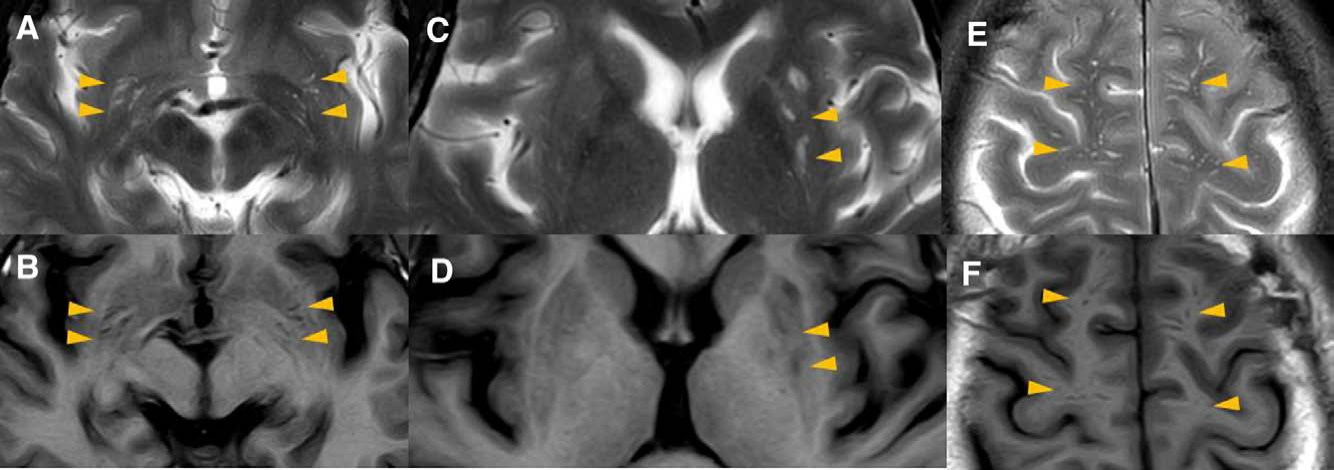
The MRI map during hospitalization. (A–F) Enlarged perivascular spaces in bilateral basal ganglia and perivascular spaces in bilateral centrum semiovale on T2WI and T1WI (yellow arrow heads). MRI = magnetic resonance imaging, T1WI = T1-weight, T2WI = T2-weight.

**Figure 3.**
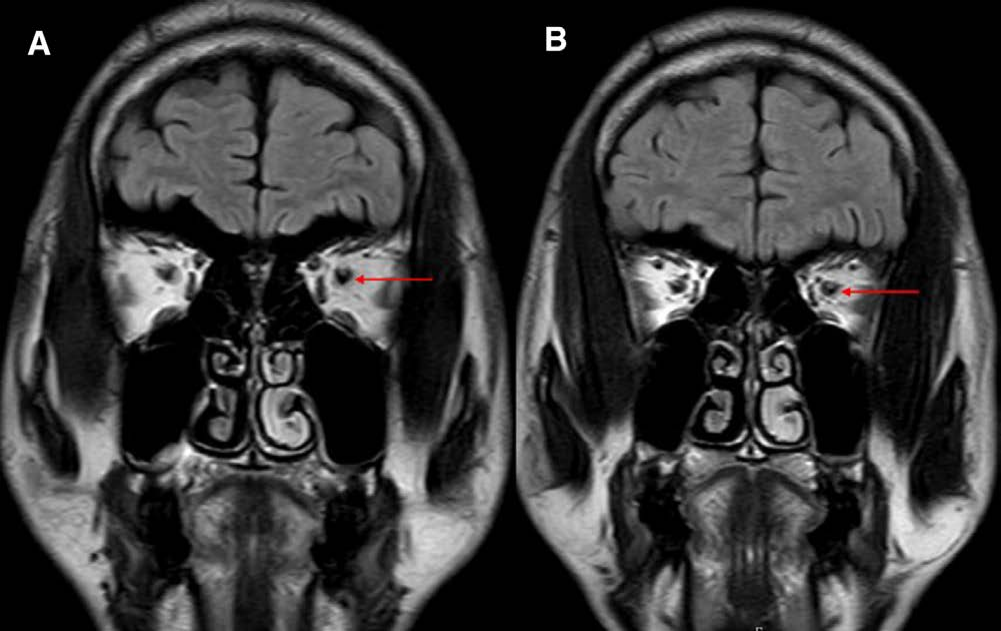
The MRI map during hospitalization. The dilatation of the optic nerve sheaths on coronal FLAIR images (red arrows). FLAIR = fluid-attenuated inversion recovery, MRI = magnetic resonance imaging.

**Figure 4.**
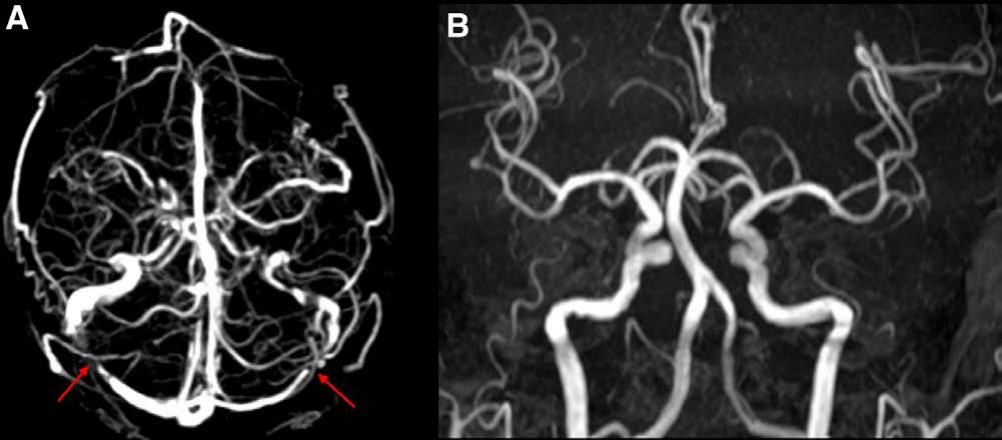
The TOF-MRV map at admission and the TOF-MRA map during hospitalization. (A) The flow void signal of bilateral transverse sinus at admission (red arrow). (B) Normal blood flow in the cerebral artery after admission. TOF-MRA = time-of-flight-magnetic resonance angiography, TOF-MRV = time-of-flight-magnetic resonance venography.

**Figure 5.**
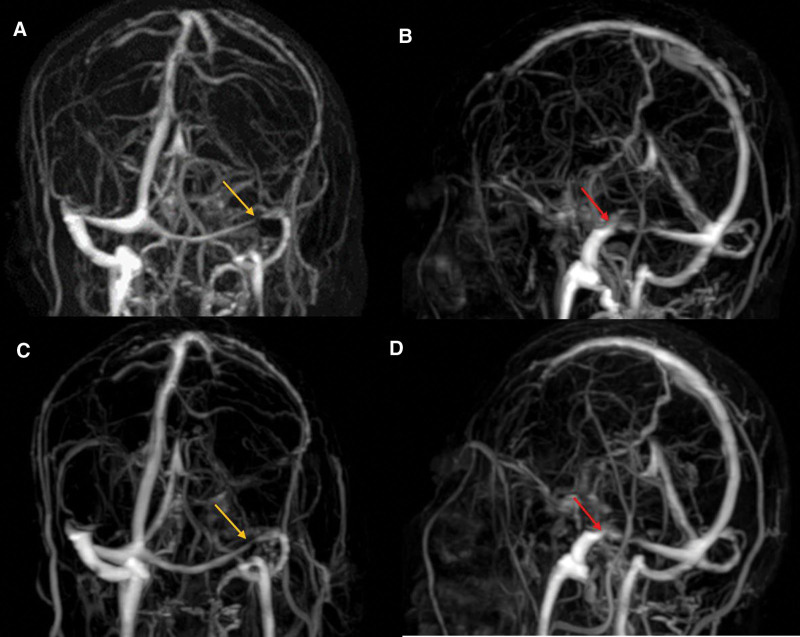
The CE-MRV map during hospitalization and the 3-month follow up CE-MRV map. (A) Hypoplasia of left transverse sinus (yellow arrow) at admission. (B) Right transverse sinus stenosis (red arrow). (C) Hypoplasia of left transverse sinus (yellow arrow) at hypoplasia of left transverse sinus (yellow arrow) at 3-month follow up. (D) Unimprovement of stenosis at right transverse sinus at 3-month follow up (red arrow). CE-MRV = contrast-enhanced magnetic resonance venography.

**Figure 6.**
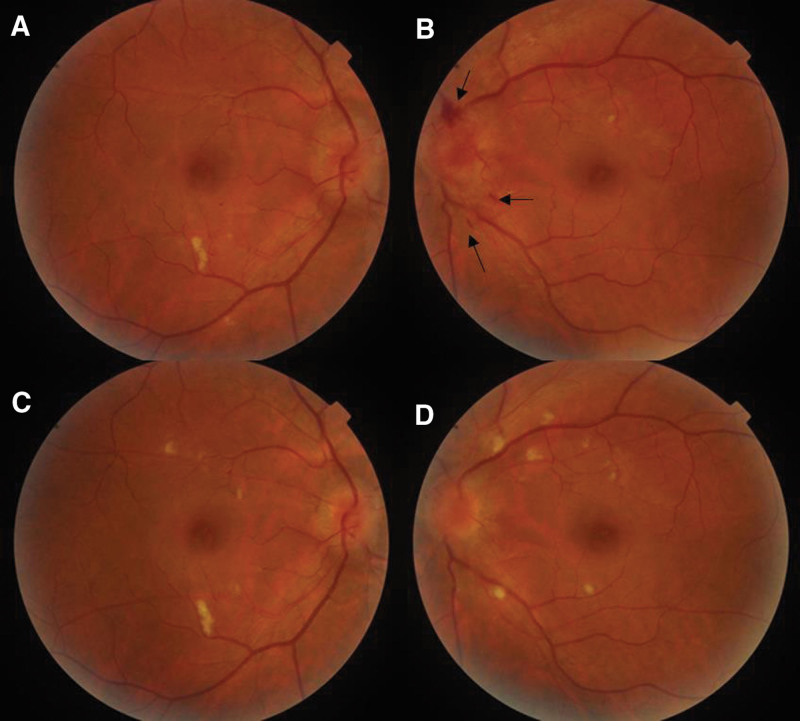
The color fundus photographs of both eyes, taken at admission and 1-month follow up. (A and B) Bilateral prominent optic disc edema, with small retinal hemorrhages around optic disc in the left eye (black arrows). (C–D) The lessened optic disc edema and disappeared retinal hemorrhages at 1-month follow up.

After admission, a lumbar puncture was performed immediately. The cerebrospinal fluid (CSF) pressure was 330 mmH_2_O; the CSF protein, cell count, and glucose level were normal, confirming increased ICP. Magnetic resonance imaging detected recent cerebral infarction in the right centrum semiovale on diffusion-weighted images (Fig. 1B), bilateral hyperintensity surrounding the ventricular white matter on coronal fluid-attenuated inversion recovery (FLAIR) images (Fig. 1A and D), lacune-presumed vascular origin in periventricular on T1-weight, T2-weight and FLAIR images (Fig. 1E–I), and EPVS in the bilateral basal ganglia and perivascular spaces in the bilateral centrum semiovale on T1-weight and T2-weight (Fig. 2A–F), suggesting the highest possibility of CSVDs. Arteriosclerosis CSVD was diagnosed according to neuroimaging standards and cerebrovascular pathological changes.^[13,14]^ In addition, dilatation of the optic nerve sheaths was detected on coronal FLAIR (Fig. 3), a radiologic sign of IIH that may be explained by an overflow of the lymphatic CSF drainage pathway.^[7]^ Time-of-flight magnetic resonance venography and angiography showed flow void signals of the bilateral transverse sinus (Fig. 4A) and normal cerebral arteries without any stenosis (Fig. 4B), respectively. Contrast-enhanced magnetic resonance venography was performed to verify the diagnosis of transverse sinus disorders. The results demonstrated right TSS and a hypoplastic left sinus (Fig. 5A and B), indicating bilateral transverse sinus obstruction.

Laboratory examinations, including routine blood testing, urinalysis, C-reactive protein, serum glucose, liver enzymes, urea, creatinine, and protein were normal; the serum homocysteine level was 87.0 μmol/L (normal value 5–15 μmol/L), and that of folate and B12 level was 2.62 ng/mL (normal value >4.4 ng/mL) and 248 pg/mL (normal value 180925 pg/mL), respectively. Coagulation testing showed that the activated partial thromboplastin time was 38.3 seconds (normal value 2636 seconds). Prothrombin time, international normalized ratio, D-dimer levels, and serum concentrations of antithrombin III, protein C, and protein S were normal. Immunological tests, including anti-cardiolipin, anti-nuclear, antikeratin, anti-SSA/Ro, anti-SSB/La, perinuclear antineutrophil cytoplasmic, cytoplasmic antineutrophil cytoplasmic antibodies, anti-SM-D1 antibody, and anti-cyclic citrullinated peptide antibody, were negative. Mutation of methylenetetrahydrofolate reductase (MTHFR) A1298C and MTHFR C677T were assessed to investigate the hyperhomocysteinemia with low folate; mutations were found for MTHFR C677T.

Based on the above findings, the patient was diagnosed with IH associated with TSS, CSVD, hyperhomocysteinemia, and hypertension. The patient was treated with mannitol injection with folate, vitamin B6, and vitamin B12. Simultaneously, antiplatelet therapy with aspirin, anti-atherosclerotic therapy with atorvastatin, and antihypertensive therapy with amlodipine were initiated. His headache was relieved gradually within 24 hours, ICP returned to normal (170 mmH_2_O) 48 hours after hospital admission, and mannitol was discontinued. Owing to symptom relief, the patient refused a digital subtraction angiography examination. LP revealed a CSF open pressure of 175 mmH_2_O after 1 week, the headaches disappeared completely, and blood pressure was well controlled. The patient was discharged from the hospital the following day and placed on aspirin, atorvastatin, amlodipine, folate, vitamin B6, and vitamin B12.

At the 1-month follow-up after discharge, fundus examination showed relief of bilateral papilledema (Fig. 6C and D). The serum homocysteine level was 27.1 μmol/L (normal value 515 μmol/L); lumbar puncture confirmed normal ICP (185 mm Hg) 3 months later. In addition, a 3-month follow-up contrast-enhanced magnetic resonance venography showed that the degree of TSS on the right side was unimproved compared to that before treatment (Fig. 5C and D). The final diagnosis was IIH, the cause of which was unrelated to TSS according to the latest criteria revision.^[15]^ At the 6-month follow-up, the patient remained asymptomatic and continued to undergo secondary prevention of ischemic stroke.

## 3. Discussion

Bilateral transverse sinus obstruction, including stenosis and thrombosis, is a causative factor for IIH, suggesting a relationship between ICP and venous sinus pressure. Previous studies showed that TSS was discerned in 23 to 93% of patients with IIH,^[16,17]^ contributing to IH with or without papilledema.^[18]^ Moreover, a minority of TSS exist in populations without elevated ICP.^[19,20]^ One study found TSS in 5% of healthy participants.^[17]^ Hence, the pathogenesis of sinus venous stenosis in patients with increased ICP remains controversial. In this case, the patient’s ICP returned to normal, and the symptoms of IH resolved after medical treatment; however, TSS persisted. This case suggests that IIH in this patient was not due to TSS or increased venous sinus pressure; the cause of TSS in IIH is unclear. The proposed cause of TSS can be divided into extraluminal and intrinsic anomalies; the former refers to a compressed venous sinus resulting from intracranial mass occupying and increased ICP, without endoluminal abnormalities; the latter includes arachnoid granulations (AG), cerebral venous sinus aplasia or septa, and venous thrombosis.^[21,22]^ The patient’s lowered ICP after lumbar puncture indicated that the most effective treatment was LP, and the etiology of TSS may be extraluminal anomalies; confirming the etiology requires further examination.

Since the pathophysiological mechanisms of IIH are still unknown, dysregulation of ICP is an important factor in IIH pathogenesis. Disorders of CSF dynamics disorders may involve CSF overproduction at the choroid plexus, CSF outflow disturbance at the AG, and increased venous sinus pressure gradients.^[1]^ Factors associated with CSF hypersecretion signs, including choroid plexus papilloma, hydrocephalus, and ventricular enlargement, were absent in this patient; TSS and increased venous sinus pressure were not the causes of IIH. Therefore, we emphasize that the etiology of IIH should be considered in cases of CSF outflow obstruction. Moreover, there has been an active debate regarding the CSF outflow pathway in recent years. Some studies have revealed that the glymphatic system is involved in this process.^[7,23]^ The glymphatic system is a brain-wide network for fluid transport and metabolic waste clearance that connects the cerebrovascular system and the CSF circulation, consisting of periarterial influx (glymphatic influx) of the subarachnoid CSF into the brain interstitium and the efflux of ISF with accompanying large-caliber veins.^[24]^ The CSF in the subarachnoid spaces enters the periarterial spaces and is transported from the cortex toward the deep white matter along the arterioles. Subsequently, the CSF is driven from the periarterial space to the brain parenchyma.^[25]^ CSF movement from the periarterial space to the brain parenchyma is mediated by AQP-4, a water channel transporter expressed in astrocytic end-feet ensheathing the brain vasculature. CSF movement toward the brain parenchyma pushes the convective ISF into the perivenous spaces surrounding the large cortical veins that empty into sinus-associated cisternal compartments.^[24]^ Consequently, CSF resorption includes 2 CSF outflow pathways: the venous and lymphatic outflow pathways.^[7]^

The venous outflow pathway is the classical CSF movement pathway. AGs in this pathway are recognized as the main pathway of CSF resorption from the subarachnoid space to the cerebral veins. When the AG fails to reabsorb CSF or is excessively enlarged to obstruct venous flow, leading to severe venous sinus stenosis, it could cause increased ICP. In addition, some meningeal inflammatory diseases can cause damage to the AG, resulting in impaired CSF absorption and IH.^[26]^ In this patient, there was no sign of significant functional impairment of the AG leading to IH, indicating that the decreased venous outflow pathway was not the cause of IIH. The lymphatic outflow pathway has gained increasing attention in recent years. Lymphatic vessels have been noninvasively confirmed in human brains using magnetic resonance imaging.^[27]^ Recent studies have demonstrated that CSF and ISF translocate into lymphatic vessels in the dural sinuses and perineural sheaths of cranial and spinal nerves, suggesting that lymphatic vessels are implicated in CSF (or ISF) drainage from the glymphatic system.^[28,29]^

Moreover, another study has shown that lymphatic vessels can directly reabsorb CSF from the subarachnoid space into the dural sinus lymphatics (meningeal lymphatics) and regulate ICP.^[30]^ Subsequently, the brain’s lymphatic system forms a drainage network extending from the dural sinuses to both eyes, tracking above the olfactory bulb and following the dural arteries and veins into the dura mater.^[28,30]^ The dural lymphatics (meningeal lymphatics) penetrating the skull base remove the CSF from the sheaths surrounding the cranial and spinal nerves, joining the deep cervical lymph nodes. Finally, the CSF is discharged into the deep cervical lymph nodes and systemic lymphatic circulation. Overflow of the lymphatic outflow pathway may be associated with excess CSF in the sheaths surrounding the cranial nerves in IIH, including the optic nerve and olfactory bulbs.^[3,5]^ Neuroimaging findings revealed dilatation of the optic nerve sheaths, suggesting dysfunction of the lymphatic systems of the brain. Furthermore, some radiographic features of CSVD, including EPVS, indicate impairment of glymphatic systems.^[31,32]^ Evidence has demonstrated the involvement of glymphatic and lymphatic systems in multiple central neurological diseases such as Parkinson disease, Alzheimer disease, CSVD, and traumatic brain injury.^[33,34]^

CSVD is a heterogeneous set of disorders with diverse etiologies, various cerebrovascular mechanisms, multifactorial pathologies,^[35]^ and multiple clinical presentations, including stroke, cognitive impairment, dementia, physical disability, and depression.^[14]^ Our understanding of the potential physiopathological mechanisms of CSVD is still limited; recent studies have shed light on the role of the glymphatic and meningeal lymphatic systems in CSVD.^[36]^ Impairment of the glymphatic system and lymphatic CSF drainage has been proven to be associated with cerebrovascular risk factors strongly linked to CSVD, including aging, hypertension, diabetes, and lipid metabolism. This condition supports the potential role of glymphatic and lymphatic system dysfunction in CSVD.^[36]^ In addition, hypertension, a significant risk factor for CSVD, can cause decreased glymphatic function.

Furthermore, some studies have revealed impaired glymphatic transport of CSF tracers in spontaneously hypertensive rats compared to normotensive rats, suggesting that hypertension could impair glymphatic function at an early age.^[32,37]^ In addition, one study showed that EPVS decreased the expression of polarized AQP4, and impaired glymphatic transport coexisted in spontaneously hypertensive rats,^[32]^ showing that decreased AQP4 polarization and EPVS may be related to impaired glymphatic function in SHRs. This finding supported the hypothesis that an impaired lymphatic system may be implicated in the pathogenesis of arteriolosclerotic CSVD. The patient had signs of EPVS and dilatation of the optic nerve sheaths, with a history of poorly-controlled hypertension, indicating that hypertension may be related to the impairment of the glymphatic and lymphatic systems.

Perivascular spaces are ISF-filled pathways around the small vessels in the brain, including the periarteriolar, pericapillary, and perivenular spaces.^[38]^ They are involved in fluid transport, CSF-ISF exchange, and drainage of waste products from the brain, which are a part of the glymphatic systems.^[31]^ Anatomically, the perivascular spaces are invested by the vessel wall at the inner boundary and by astrocytic end feet and pia matter on the exterior. Furthermore, AQP4, highly polarized at the end-feet of astrocytes, facilitates the transport of CSF from perivascular spaces into the ISF space and active flushing of ISF^[24]^; this supports the crucial role of astrocytes in the glymphatic system.^[39]^ Polarized AQP4 on perivascular astrocytic end feet is a strong regulator of normal glymphatic function.^[40]^ Additional evidence of the aberrant location of AQP4 on astrocyte end feet and damage was related to cognitive dysfunction, which can be described in animal models of CADASIL. This is followed by abnormally organized AQP4 expression in the end-feet of astrocytes, supporting the idea of loss of astrocytic coverage of the vasculature in CADASIL.^[38,41]^

Moreover, one study demonstrated significant astrocytic end-foot disruptions and dislocalization of the AQP4 channel in the HHcy model, suggesting that astrocyte dysfunction is associated with hyperhomocysteinemia.^[42,43]^ However, the involvement of the glymphatic system in this process has not been described; further investigations reporting the roles of astrocytes and hyperhomocysteinemia in the glymphatic system are required.^[31]^ This patient showed high homocysteine levels, and mutations were found in MTHFR C677T, exacerbating the disruption of the astrocytic end-foot.

## 4. Conclusion

The glymphatic system and lymphatic drainage of CSF may serve as a compensatory mechanism for regulating increased ICP in the patient with bilateral venous sinus obstruction, indicating an impaired venous outflow pathway, possibly associated with dysfunction of the glymphatic and lymphatic systems in patients with CSVD.

## Author contributions

WL got the research ideas and drafted the article. LJ communicated with patient and his family.

**Data curation:** Fengbing Yang, Hongjiang Cheng, Huimin Li, Dandan Zhang, Yan Liu.

**Methodology:** Jing Hou.

**Software:** Wei Liu.

**Supervision:** Longbin Jia, Lina Xu.

**Writing – original draft:** Wei Liu.

**Writing – review & editing:** Wei Liu.
